# Clinical spectrum and complications of *Plasmodium vivax* malaria: A retrospective study from Delhi, India

**DOI:** 10.5281/zenodo.17120057

**Published:** 2025-09-15

**Authors:** Dharmander Singh, Azhar Uddin, Kanupriya Bajaj, Arushi Chaturvedi, Radhika Garg, Kailash Chandra, Ayan Das, Sunil Kohli, Vineet Jain

**Affiliations:** 1 Department of Medicine, Hamdard Institute of Medical Sciences and Research (HIMSR), Delhi, India.; 2 Department of Community Medicine, Hamdard Institute of Medical Sciences and Research (HIMSR), Delhi, India.; 3 Department of Biochemistry, Hamdard Institute of Medical Sciences and Research (HIMSR), Delhi, India.; 4 Department of Microbiology, Hamdard Institute of Medical Sciences and Research (HIMSR), Delhi, India.; 5 Department of Emergency Medicine, Hamdard Institute of Medical Sciences and Research (HIMSR), Delhi, India.

## Abstract

**Background:**

*Plasmodium vivax* malaria, traditionally regarded as benign, is now recognised to cause severe illness. India bears a high burden of *P. vivax* malaria, yet data on its clinical spectrum and severity predictors remain limited. This study aimed to describe the clinical and laboratory profile of *P. vivax* malaria and identify risk factors for severe disease in a tertiary care setting.

**Materials and Methods:**

A retrospective study of 361 patients diagnosed with *P. vivax* malaria between June 2020 and May 2024 was conducted at a tertiary hospital in South Delhi, India. Diagnosis was confirmed by peripheral smear and/or rapid diagnostic tests. Patients were categorised into complicated and uncomplicated groups using WHO criteria. Demographic, clinical, and laboratory data were analysed with chi-square test, odds ratios, correlation analysis, and logistic regression.

**Results:**

Of 361 patients, 167 (46.3%) had complications. Mean age was 31 years with male predominance (64.6%), though complications were more frequent in females (42% vs. 32%, P=0.039). Anaemia (73.4%), thrombocytopenia (57.9%), and leucocytosis were common. Thrombocytopenia (OR 3.20, P<0.001) and leucocytosis (OR 2.37, P<0.05) were significantly linked to severity. Elevated creatinine (OR=6.07, P 0.001) and hyperbilirubinemia (OR=3.71, P<0.001) strongly correlated with complications. Breathlessness and pleural effusion were also more common in severe cases. Strong associations were observed between anaemia and hyperbilirubinemia (r 0.75), bleeding and ARDS (r 0.82), and mortality with shock (r=0.74).

**Conclusion:**

Nearly half of *P. vivax* cases developed severe complications, challenging its benign perception. Anaemia, thrombocytopenia, leucocytosis, and organ dysfunction were key severity markers. Higher complication rates in females and afebrile cases highlight diagnostic and social challenges. Early recognition of atypical features and vigilant monitoring are crucial to improve outcomes in endemic regions.

## Introduction

Malaria is a disease caused by *Plasmodium* parasites and is transmitted through the bite of infected female *Anopheles* mosquitoes, which continues to be a major global health concern. The World Health Organization (WHO) identifies five species that infect humans: *P. falciparum*, *P. vivax*, *P. ovale* (subdivided into *wallickeri* and *curtisi*), *P. malariae*, and *P. knowlesi* [1,2]. Of these, *P. falciparum* and *P. vivax* are the most common and significant, with *P. falciparum* historically linked to severe malaria cases [1,3]. Despite an ongoing malaria control programme, India documented approximately 3.34 million malaria cases in 2022 as per the WHO’s World Malaria Report 2023. Notably, India was the only country in the WHO South-East Asia Region to avoid a rise in malaria mortality between 2020 and 2022 [4]. This highlights the critical need for sustained research and public health efforts.

Traditionally, *P. vivax* has been regarded as a less severe species compared to *P. falciparum*. However, emerging evidence contradicts this existing knowledge, demonstrating that *P. vivax* also leads to severe complications similar to those caused by *P. falciparum*, such as acute kidney injury, jaundice, thrombocytopenia, sepsis, severe haemolysis, pulmonary oedema, cerebral malaria, shock, and even death [5-9]. These observations indicate that *P. vi-vax* malaria has a broad clinical spectrum, from uncomplicated cases to severe, potentially fatal outcomes [9]. Despite its prevalence and impact in India, there remains a paucity of comprehensive studies exploring the clinical profile and complications of *P. vivax* malaria in this population [10].

This study aimed to bridge this knowledge gap by exploring the clinical presentations and laboratory characteristics of *P. vivax* malaria in a cohort of over 350 patients at a tertiary care hospital in South Delhi. By analysing both complicated and uncomplicated cases, the research intends to enhance the understanding of the disease’s severity and strategise to improve its management. Given the high burden of *P. vivax* in India, this work is poised to contribute practical, evidence-based insights to reduce associated complications and mortality.

## Materials and Methods

This retrospective study was carried out between June 2020 and May 2024 in the Department of Medicine at a tertiary care hospital in South Delhi, India.

*Plasmodium vivax* malaria was diagnosed using either peripheral smear (using Giemsa stain) or malaria antigen rapid diagnostic test. Patients infected with more than one pathogen, or those with localised infections evident on clinical or laboratory evaluation (such as liver abscess, urinary tract infection/pyelonephritis, pneumonia with mucopurulent expectoration, or acute tonsillitis), were excluded from the study. Initially, a total of 429 patients were considered for the study having tested positive for *P. vivax* infection. Of these, 68 were excluded due to reasons such as denial of consent, unavailability of complete data, mixed infections with *P. falciparum*, dengue etc., or the patient leaving the hospital prematurely against medical advice, resulting in 361 patients that were included in the study.

A detailed history was taken for all subjects and a thorough physical examination was conducted. All the individuals included in the study were subjected to complete haemogram (using Sysmex Haematology analyser), liver function test (LFT), kidney function test (KFT) (using Siemens Xpand biochemistry autoanalyser), abdominal ultrasonography (using Samsung HS70 5D) and chest radiograph (using Siemens Multiselect DR) and wherever indicated, further investigations included Arterial Blood Gas analysis (ABG), PT-INR/ APTT (using Sysmex Haematology Analyser), urine routine and microscopy (using Automated Urine Chemistry & Microscopy Analyser), serum G6PD levels (using kinetic UV method), hepatitis B surface antigen and hepatitis C Virus antibody (using Rapid immunochromatographic test), glycated haemoglobin (HbA1c) (by HPLC method), CT chest, and CT/MRI brain (using Siemens 1.5 T MRI Machine and a Siemens 16 S CT Machine); few additional tests were performed as clinically indicated. The patients’ platelet count and liver function tests were checked and recorded daily. For the haemoglobin levels, platelet count, serum sodium levels and serum potassium levels, the lowest value during the course of the hospital stay were recorded. In the case of blood urea, serum creatinine, serum bilirubin, serum glutamic oxaloacetic transaminase (SGOT) and serum glutamic pyruvic transaminase (SGPT), the highest values were recorded of each patient during the course of hospital stay. The measurement of haematological and biochemical parameters was done at the diagnostic laboratory of the hospital. The standardisation of these parameters was achieved by using internal and external quality control samples, and the relevant details were documented in the laboratory. The reference ranges of the investigations carried out were defined by the reference ranges of the hospital laboratory.

The complications that were included in the study were:

1. Severe anaemia: Defined as Hb <7g/dl;
2. Hyperbilirubinemia: Defined as total serum bilirubin > 3g/dl;
3. AKI: Defined as serum creatinine > 3mg/dl;
4. Shock: Defined as Systolic BP <90mmHg &/or Diastolic BP <60mmHg even after adequate IV fluid resuscitation (at the rate of 30ml/kg);
5. Neurological complications: Defined by presence of delirium, encephalopathy, comatose state or seizure;
6. Metabolic acidosis: Defined as pH <7.35;
7. Bleeding: Active bleed from any site or presence of melena;
8. ARDS: Defined as acute onset respiratory failure with chest radiogram finding of bilateral pulmonary infiltrates with severe hypoxemia without any presence of cardiogenic pulmonary oedema [8]
9. Mortality

Patients with one or more of the above-mentioned complications were included in the complicated group and in the absence of any of these complications, were included in the uncomplicated group.

The study was carried out in accordance with the Basic Principles defined in ICS (9 May 1997) ‘Guidance for good clinical practice’ and the principles enunciated in Declaration of Helsinki (Edinburgh, October 2000). Informed consent was obtained from all subjects before their participation in the study and any identifying information and data was kept confidential. Ethical clearance was obtained from the Institutional Ethics Committee.

All patients were managed according to the WHO standard treatment guidelines for *P. vivax* malaria. Each patient received chloroquine for the acute infection, followed by primaquine for radical as mean ± standard deviation (SD), while categorical variables were presented as frequencies and percentages. For comparisons between complicated and uncomplicated groups, two-sample independent t-tests were applied for continuous variables. For categorical variables, Chi-square test or Fisher’s exact test was used as appropriate (when expected cell counts were <5). Logistic regression was employed to estimate odds ratios (OR) with 95% confidence intervals (CI) for predictors of severity. Relative risk (RR) with 95% CI was also calculated where relevant. Correlation between complications was assessed using Pearson’s correlation coeffcient. A p-value <0.05 was considered statistically significant.

## Results

This retrospective study included 361 patients with *P. vivax* malaria, with 167 (46.3%) in the complicated group and 194 (53.7%) in the uncomplicated group. As per Table 1, the mean age was similar across groups (32.10 12.35 years in complicated vs. cure. The treatment protocol was uniform across both complicated and uncomplicated groups; therefore, treatment outcomes were not separately analysed.

**Table 1:** Demographic data of individuals included in the study and their distribution in the two groups.

Characteristics of the participants	Complicated group (N=167) (%)	Uncomplicated group (N=194) (%)	Total study population (N=361) (%)	P-value
Age (Mean ± SD)*	32.10 ±12.35	30.87 + 12.84	31.44 + 12.61	0.356
Male	97 (42.17)	133 (57.83)	230 (100)	0.039
Female	70 (53.44)	61 (46.56)	131 (100)	
Hypertension	2 (66.67)	1 (33.33)	3 (100)	0.442
Type 2 DM	4 (30.77)	9 (69.23)	13 (100)	
Hypothyroidism	5 (55.56)	4 (44.44)	9 (100)	

* Two sample independent t-test was used for analysing age differences. Chi-square and Fisher's Exact tests were used for analysing gender and comorbidity differences. P-value <0.05 is considered significant.

### Data analysis

All collected data were compiled in Microsoft Excel 2019 and analysed using IBM SPSS Statistics version 2020. Continuous variables were summarised 30.87±12.84 years in uncomplicated; P=0.287), with an overall male predominance (64.63% male, 35.37% female). However, the complicated group had a significantly higher proportion of females (42% vs. 32% in the uncomplicated group; P= 0.039). Comorbidities were infrequent and evenly distributed.

Table 2 shows that fever was absent in 6% of complicated and 13% of uncomplicated cases, while chills were absent in 10% and 18%, respectively. Breathlessness (20.96% vs. 2.06%, P<0.001) and pleural effusion (5.99% vs. 0.52%, P=0.004) were significantly more prevalent in the complicated group. Cough (19.67% vs. 16.49%, P=0.421) and hepatosplenomegaly (similar in both groups) showed no association with severity.

**Table 2:** Occurrence of clinical features observed in patients with and without severe complications.

Clinical characteristic	Occurrence in patients with severe complications (N=167) (%)	Occurrence in patients without severe complications (N=194) (%)	Total occurrence (N=361) (%)
Fever	157 (94.01)	169 (87.11)	326 (90.30)
Chills	149 (89.22)	158 (81.44)	307 (85.04)
Body ache	81 (48.50)	112 (57.73)	112 (31.02)
Headache	96 (57.48)	110 (56.70)	206 (57.06)
Cough	33 (19.67)	32 (16.49)	65 (18.01)
Breathlessness	35 (20.96)	4 (2.06)	39 (10.80)
Pleural effusion	10 (5.99)	1 (0.52)	11 (3.05)
Hepato-splenomegaly	74 (44.31)	71 (36.60)	145 (40.17)

Table 3 and Table 4 show that laboratory analyses revealed anaemia in 79.04% of complicated vs. 68.56% of uncomplicated patients (OR=1.73, P= 0.024), with lower mean haemoglobin in the complicated group (P<0.001). Thrombocytopenia was nearly twice as common in the complicated group (OR 3.20, P<0.001), as was leucocytosis (OR 2.37, P 0.05), while leukopenia showed no association (OR 1.02). Hyponatremia was slightly more frequent in complicated cases (60.48% vs. 56.19%, P= 0.409), but hypokalaemia was similar across groups (32.93% vs. 35.57%, OR 0.89). Elevated serum creatinine (OR 6.07, P<0.001) and urea (OR 5.25, P<0.001) were strongly linked to complications, as were hyperbilirubinemia (OR 3.71, P<0.001) and elevated SGOT (OR=2.16, P 0.001), though SGPT and ALP showed weaker associations.

**Table 3:** Blood data from patients with (N=167) or without severe complications (N=194).

Characteristic	With severe complications (%)	Without severe complications (%)	Total occurrence (%)	P-value*	RR [95% Cl]	OR [95% Cl]
*Haemoglobin*						
≥12g / dL	35 (20.96)	61 (31.44)	96 (26.59)	0.024	1.15 [1.02,	1.73 [1.07,
<12 g / dL)	132 (79.04)	133 (68.56)	265 (73.41)		1.30]	2.79]
*Total leucocyte count*						
<4000 cells / mm^3^	68 (40.72)	78 (40.21)	146 (40.44)	0.921	1.01 [0.79,	1.02 [0.67,
≥4000 cells / mm^3^	99 (59.28)	116 (59.79)	215 (59.56)		1.30]	1.56]
*Total leucocyte Count*						
>11000 cells / mm^3^	6 (3.59)	3 (1.55)	9 (2.49)	0.312	2.32 [0.59,	2.37 [0.64,
≤ll000 cells / mm^3^	161 (96.41)	191 (98.45)	352 (97.51)		9.15]	8.78]
*Platelet Count*						
≥0.5 x 10^5^ mm^3^	63 (37.72)	128 (65.98)	191 (52.91)	0.000	1.83 [1.46,	3.20 [2.08,
<0.5 x 10^5^ mm^3^	104 (62.28)	66 (34.02)	170 (47.03)		2.30]	4.92]
*Serum creatinine*						
≤1.2 mg / dL	103 (61.68)	176 (90.72)	279 (77.29)	0.000	4.13 [2.56,	6.07 [3.43,
>1.2 mg / dL	64 (38.32)	18 (9.28)	82 (22.71)		6.68]	10.76]
*Blood urea*						
≤40 mg / dL	94 (56.29)	169 (87.11)	263 (72.85)	0.000	3.39 [2.26,	5.25 [3.13,
>40 mg / dL	73 (43.71)	25 (12.89)	98 (27.15)		5.08]	8.79]
*Serum sodium*						
≥135mmol / L	66 (39.52)	85 (43.81)	151 (41.83)	0.409	1.08 [0.90,	1.19 [0.78,
<135 mmol / L	101 (60.48)	109 (56.19)	210 (58.17)		1.28]	1.82]
*Serum potassium*						
≥3.5mmol / L	112 (67.07)	125 (64.43)	237 (65.65)	0.599	0.92 [0.69,	0.89 [0.58,
<3.5 mmol / L	55 (32.93)	69 (35.57)	124 (34.35)		1.23]	1.37]
*Total bilirubin*						
≤1.2 mg / dL	30 (17.96)	87 (44.85)	117 (32.41)	0.000	1.49 [1.29,	3.71 [2.29,
>1.2 mg / dL	137 (82.04)	107 (55.15)	244 (67.59)		1.78]	6.02]
*SGOT serum*						
≤45 U / L	86 (51.50)	135 (69.59)	221 (61.22)	0.000	1.59 [1.22,	2.16 [1.40,
>45 U / L	81 (48.50)	59 (30.41)	140 (38.78)		2.08]	3.31]
*SGPT serum*						
≤45 U / L	89 (53.29)	116 (59.79)	205 (57.26)	0.213	1.16 [0.92,	1.30 [0.86,
>45 U / L	78 (46.71)	78 (40.21)	153 (42.74)		1.47]	1.98]
*Serum ALP*						
≤110 U / L	66 (39.52)	104 (53.61)	170 (47.09)	0.007	1.30 [1.07,	1.77 [1.16,
>110 U / L	101 (60.48)	90 (46.39)	191 (52.91)		1.58]	2.69]

* Based on Chi-square or Fisher exact tests. P<0.05 is considered significant.

**Table 4:** Blood data means (±SD) from patients with (N=167) or without severe complications (N=194).

Parameter	With severe complications		Without severe complications		P-value*
	Mean	±2 SD	Mean	±2 SD	
Mean haemoglobin	9.48	2.99	10.84	2.21	0.000
Total leucocyte count	4.97	2.92	4.63	1.85	0.196
Platelet count	45.49	34.64	69.67	54.16	0.000
Serum creatinine	1.46	1.62	0.89	0.23	0.000
Serum urea	46.48	36.76	28.82	11.98	0.000
Serum sodium	133.94	4.42	134.43	4.82	0.315
Serum potassium	3.82	0.60	3.78	0.55	0.512
Total serum bilirubin	3.57	3.41	1.40	0.66	0.000
Serum SGOT	71.20	96.16	42.61	26.44	0.000
Serum SGPT	56.50	47.09	51.06	61.35	0.342
Serum ALP	142.75	67.24	120.36	52.96	0.001

* Based on paired t-tests. P<0.05 is considered significant.

Correlation analysis, shown in Table 5 highlighted strong positive relationships, including anaemia with hyperbilirubinemia (r=0.75) and AKI (r 0.69), bleeding with ARDS (r 0.82), neurological complications with shock (r 0.79), mortality with shock (r=0.74) and acidosis (r=0.68).

**Table 5:** Correlation coefficient matrix between different clinical indicators of *P. vivax* malaria infections. P-values are shown between brackets.

Hyper-bilirubinemia	73 1.0000								
Acute kidney injury	4	9 1 0000							
	0.2801								
	(0.4654)								
Severe anaemia	16	5	43 1.0000						
	0.7522*	0.6980*							
	(0.0194)	(0.0366)							
Neurological complication	3	0	1	16 1.0000					
	-0.1087	-0.6802*	-0.4783						
	(0.7807)	(0.0438)	0.1928						
Bleeding	9	0	5	3	36 1.000				
	0.7217*	-0.2356	0.4348	0.3261					
	(0.0281)	(0.5417)	(0.2422)	(0.3918)					
ARDS**	9	1	4	2	5	22 1.0000			
	0.6839*	0.0656	0.4616	0.0299	0.8292*				
	(0.0422)	(0.8669)	(0.2110)	(0.9391)	(0.0057)				
Metabolic acidosis	4	2	4	3	1	0	8 1.0000		
	0.2348	0.1956	0.1391	0.2739	-0.1087	-0.3718			
	(0.5431)	(0.6140)	(0.7211)	(0.4757)	(0.7807)	(0.3244)			
Shock	10	0	4	6	5	3	4	24 1.0000	
	0.2790	-0.5881	-0.1931	0.7941*	0.4636	0.1013	0.3820		
	(0.4672)	(0.0958)	(0.6185)	(0.0106)	(0.2088)	(0.7955)	(0.3103)		
Mortality	4	0	2	2	1	0	3	5	5 1.0000
	0.1854	-0.3834	-0.2371	0.4311	0.0129	-0.3517	0.6811*	0.7404*	
	(0.6330)	(0.3083)	(0.5391)	(0.2467)	(0.9737)	(0.3533)	(0.0434)	(0.0225)	
	Hyper-bilirubinemia	Acute kidney injury	Severe anaemia	Neurological complication	Bleeding	ARDS	Metabolic acidosis	shock	Mortality

* P<0.05 is considered significant. ** ARDS: Acute Respiratory Distress Syndrome.

## Discussion

This study provided compelling evidence of the significant burden of complications associated with *P. vivax* infections, which was traditionally regarded as benign. In our study, 46.3% of patients had complications and our findings align with the reported range of 30% to 60% in previous studies [5,12], thus reinforcing the impression that *P. vivax* malaria poses a substantial clinical challenge in endemic countries and regions of South East Asia and Africa. The statistical comparison of the clinical and laboratory profiles of complicated and uncomplicated cases offered practical insights into disease presentation, severity markers, and management considerations. These findings challenge outdated perceptions and emphasise the need for heightened clinical awareness and proactive diagnostic strategies.

The demographic characteristics of this study lays a foundation for understanding the epidemiology of *P. vivax* malaria. The mean age of approximately 31 years across both complicated and uncomplicated groups is consistent with previous studies by Matlani *et al.* [5] and Yadav *et al.* [12], suggesting that younger adults are more prone, likely due to increased outdoor exposure or occupational risk in endemic areas. The male-to-female ratio of 1.76:1 echoes patterns observed in multiple studies [5,14,15], often attributed to greater mosquito exposure among males. However, a striking observation in our study is the significantly higher proportion of complicated cases among females (42% vs. 32%, P 0.039). This gender disparity is not widely documented in *P. vivax* literature and may reflect socioeconomic factors unique to India, such as delayed healthcare-seeking behaviour among women due to financial constraints, undernourishment, or limited access to education leading to more risk of complications in females. This finding calls for further analysis to elucidate its causes and implications, potentially informing targeted public health interventions for vulnerable populations.

Clinical manifestations in our study revealed both typical and few unexpected observations, enriching our understanding of *P. vivax* presentation. Fever with chills and rigours, a classic hallmark of malaria [16], was absent in 6% of complicated and 13% of uncomplicated cases, while chills were missing in 10% and 18% of these groups, respectively. These observations bolster the concept of ‘afebrile malaria’, previously noted by Neeshu *et al.* [17], and our larger sample size enhances its clinical significance. Notably, 8% of afebrile cases developed complications, suggesting active disease progression despite the absence of fever, possibly due to late presentation or immune modulation. This challenges the traditional reliance on fever for diagnosis and highlights the need for evaluation of malaria in endemic areas with a low index of suspicion, even in the absence of typical symptoms, to ensure prompt diagnosis and treatment.

Beyond fever, non-specific symptoms like headache and body ache were prevalent but did not distinguish between complicated and uncomplicated cases, serving as general markers of infection rather than severity indicators. Cough, observed in 19.67% of complicated and 16.49% of uncomplicated cases (P=0.421), likely reflects red blood cell (RBC) lysis rather than a precursor to acute respiratory distress syndrome (ARDS), differing from studies linking respiratory symptoms to severe malaria [8]. This discrepancy may stem from our larger cohort or regional differences in disease expression. Hepatosplenomegaly was equally common in both groups, consistent with the parasite’s hepatic and splenic lifecycle [18], contrasting with Matlani *et al.* [5], who reported higher rates in complicated cases, possibly due to their inclusion of paediatric patients. Conversely, breathlessness and pleural effusion were significantly more frequent in the complicated group (P 0.001 and P=0.004, respectively), aligning with prior reports [5,8] and highlighting pulmonary involvement as a key feature of severe disease, necessitating close respiratory monitoring.

Laboratory parameters offered critical insights into the pathophysiological mechanisms driving *P. vivax* malaria complications. Anaemia affected 73.41% of the cohort, with a higher prevalence in the complicated group (79.04% vs. 68.56%, OR=1.73, P 0.024), corroborating findings by Matlani *et al.* [5] and Rahimi *et al.* [7]. This is likely multifactorial, resulting from RBC destruction, parasite-mediated haemoglobin digestion, and splenic sequestration [27,28,29]. Lower mean haemoglobin levels in complicated cases emphasise haemolysis as a driver of severity. Leucocytosis emerged as a significant marker of complications (OR 2.37, P<0.05), potentially reflecting higher parasitaemia or an exaggerated inflammatory response [30]. This extends observations by Wynberg *et al.* [30] of variable leukocyte counts in malaria and positions leucocytosis as a novel prognostic tool in *P. vivax*, distinguishing it from leukopenia, which showed no association with severity (OR 1.02). These haematological shifts suggest that admission leukocyte counts could guide risk stratification and management decisions.

Thrombocytopenia was markedly more prevalent in the complicated group (OR=3.20, P 0.001), aligning with extensive literature [7,8,31,32] and reflecting mechanisms such as immune-mediated destruction, oxidative stress, and platelet sequestration [32,33,34]. Platelets’ antiparasitic role [35] may further link lower counts to increased severity, reinforcing thrombocytopenia as a critical indicator requiring vigilant monitoring. Electrolyte imbalances presented nuanced findings: hyponatremia was slightly more common in complicated cases (60.48% vs. 56.19%, P 0.409) but lacked statistical significance, differing from studies associating it with severe malaria [19,21,23]. No hyperkalaemia was observed despite haemolysis and renal risks, while hypokalaemia was frequent across both groups (32.93% vs. 35.57%, OR=0.89) without correlating with severity. Two rare cases of hypokalaemic paralysis, previously reported in severe malaria [20], highlight the need for potassium monitoring, though hypokalaemia itself did not elevate complication risk in our cohort.

Renal and hepatic dysfunction significantly contributed to disease severity. Elevated serum creatinine (OR 6.07, P 0.001) and urea (OR 5.25, P< 0.001) strongly correlated with complications, likely due to haemolysis-induced acute tubular necrosis [24], consistent with Khan *et al.* [7]. However, our analysis suggests that acute kidney injury (AKI) did not predispose to mortality, implying potential reversibility with prompt intervention—a more optimistic outlook than previously considered. Hepatic involvement was equally prominent, with hyperbilirubinemia (OR 3.71, P 0.001) and elevated SGOT (OR 2.16, P<0.001) tied to complications, reflecting haemolysis and liver stress [7,8,25]. SGPT and ALP showed weaker associations, indicating that while hepatic dysfunction is common, it may not be a primary mortality driver in *P. vivax*.

Correlation analyses illuminated the interplay between complications. Anaemia strongly correlated with hyperbilirubinemia (r 0.75) and AKI (r=0.69), underscoring haemolysis as a unifying mechanism. Bleeding exhibited a robust association with ARDS (r=0.82), suggesting microvascular damage as a shared pathway, as proposed by Jain *et al.* [8]. Neurological complications linked closely with shock (r 0.79), while mortality correlated with shock (r 0.74) and acidosis (r=0.68), consistent with severe malaria patterns [6,9]. These relationships highlight the multifactorial nature of *P. vivax* complications, necessitating comprehensive monitoring of haematological, renal, hepatic, and respiratory parameters to mitigate adverse outcomes.

Our 46.3% complication rate falls within the meta-analysis spectrum reported by Rahimi *et al.* [6], though our large Indian cohort adds regional context. Anaemia and thrombocytopenia trends align with Matlani *et al.* [5], but leucocytosis as a severity predictor is a novel contribution, extending beyond prior literature. Unlike Khan *et al.* [7], who emphasised renal complications’ gravity, our data suggest AKI’s reversibility, offering hope for better outcomes. Pulmonary findings, including breathlessness and pleural effusion, echo Jain *et al.* [8], while afebrile malaria with complications builds on Mathews *et al.* [9], challenging diagnostic norms. The higher complication rate among females and leucocytosis as a marker of severity are unique observations, potentially reflecting social barriers and inflammatory dynamics, respectively, and warrant further exploration.

### Strengths and limitations

This study’s strengths lie in its large sample size of over 350 patients, enhancing the reliability of our findings, and its detailed comparison of complicated and uncomplicated cases, offering actionable clinical insights. The correlation analyses provide a holistic view of complication interrelationships, enriching the discussion. However, the retrospective, single-center design limits generalisability to other regions or healthcare settings. The lack of parasite density data, inherent to the study’s retrospective nature, prevents analysis of its role in severity. Additionally, focusing solely on inpatients may have excluded milder cases, potentially inflating the complication rate. Future prospective, multicenter studies incorporating parasite load and outpatient data could overcome these limitations and broaden our understanding of *P. vivax* malaria.

## Conclusions

This study underscores that *P. vivax* malaria can result in severe complications in nearly half of cases, driven by anaemia, thrombocytopenia, leucocytosis, and renal and hepatic dysfunction. Afebrile presentations and higher complication rates among females highlight diagnostic challenges and social disparities requiring further investigation. These findings advocate for proactive management, including early recognition of atypical symptoms and thorough laboratory monitoring, to reduce morbidity in endemic regions like India.
